# The effects of tidal volume size and driving pressure levels on pulmonary complement activation: an observational study in critically ill patients

**DOI:** 10.1186/s40635-020-00356-6

**Published:** 2020-12-18

**Authors:** Friso M. de Beer, Luuk Wieske, Gerard van Mierlo, Diana Wouters, Sacha Zeerleder, Lieuwe D. Bos, Nicole P. Juffermans, Marcus J. Schultz, Tom van der Poll, Wim K. Lagrand, Janneke Horn, for the BASIC–study group, F. M. de Beer, L. D. Bos, T. A. Claushuis, G. J. Glas, J. Horn, A. J. Hoogendijk, R. T. van Hooijdonk, M. A. Huson, M. D. de Jong, N. P. Juffermans, W. K. Lagrand, T. van der Poll, B. Scicluna, L. R. Schouten, M. J. Schultz, K. F. van der Sluijs, M. Straat, L. A. van Vught, L. Wieske, M . A. Wiewel, E. Witteveen

**Affiliations:** 1 Department of Intensive Care Medicine, Amsterdam UMC, University of Amsterdam, Amsterdam, The Netherlands; 2 Laboratory of Experimental Intensive Care and Anesthesiology (LEICA), Amsterdam UMC, University of Amsterdam, Amsterdam, The Netherlands; 3 Department of Anesthesiology, Amsterdam UMC, University of Amsterdam, Mail stop H1-118,Meibergdreef 9, 1105 AZ Amsterdam, The Netherlands; 4 Department of Neurology, Amsterdam UMC, University of Amsterdam, Amsterdam, The Netherlands; 5 Department of Hematology, Amsterdam UMC, University of Amsterdam, Amsterdam, The Netherlands; 6 Department of Immunopathology, Sanquin Research and Landsteiner Laboratory, Amsterdam, The Netherlands; 7 Mahidol-Oxford Tropical Medicine Research Unit (MORU), Mahidol University, Bangkok, Thailand; 8 Nuffield Department of Medicine, University of Oxford, Oxford, UK; 9 Department of Internal Medicine, Amsterdam UMC, University of Amsterdam, Amsterdam, The Netherlands; 10 Center for Experimental and Molecular Medicine (CEMM), Amsterdam UMC, University of Amsterdam, Amsterdam, The Netherlands

**Keywords:** Intensive care, Critical care, Mechanical ventilation, Tidal volume, Driving pressure, Bronchoalveolar lavage, Complement, Complement activation, Complement component 5, C5a

## Abstract

**Background:**

Mechanical ventilation can induce or even worsen lung injury, at least in part via overdistension caused by too large volumes or too high pressures. The complement system has been suggested to play a causative role in ventilator-induced lung injury.

**Aims and methods:**

This was a single-center prospective study investigating associations between pulmonary levels of complement activation products and two ventilator settings, tidal volume (*V*_T_) and driving pressure (Δ*P*), in critically ill patients under invasive ventilation. A miniature bronchoalveolar lavage (BAL) was performed for determination of pulmonary levels of C5a, C3b/c, and C4b/c. The primary endpoint was the correlation between BAL fluid (BALF) levels of C5a and *V*_T_ and Δ*P*. Levels of complement activation products were also compared between patients with and without ARDS or with and without pneumonia.

**Results:**

Seventy-two patients were included. Median time from start of invasive ventilation till BAL was 27 [19 to 34] hours. Median *V*_T_ and ΔP before BAL were 6.7 [IQR 6.1 to 7.6] ml/kg predicted bodyweight (PBW) and 15 [IQR 11 to 18] cm H_2_O, respectively. BALF levels of C5a, C3b/c and C4b/c were neither different between patients with or without ARDS, nor between patients with or without pneumonia. BALF levels of C5a, and also C3b/c and C4b/c, did not correlate with *V*_T_ and Δ*P*. Median BALF levels of C5a, C3b/c, and C4b/c, and the effects of *V*_T_ and Δ*P* on those levels, were not different between patients with or without ARDS, and in patients with or without pneumonia.

**Conclusion:**

In this cohort of critically ill patients under invasive ventilation, pulmonary levels of complement activation products were independent of the size of *V*_T_ and the level of Δ*P*. The associations were not different for patients with ARDS or with pneumonia. Pulmonary complement activation does not seem to play a major role in VILI, and not even in lung injury per se, in critically ill patients under invasive ventilation.

## Background

Invasive ventilation has a strong potential to cause so-called ventilator-induced lung injury (VILI) [1], at least in part via overdistension of lung units due to the use of too large volumes or too high pressures [2]. Ventilation with a low tidal volume (*V*_T_) of 6 ml/kg predicted body weight (PBW) clearly improves outcomes in patients with acute respiratory distress syndrome (ARDS) [3], and maybe also in patients without ARDS [4, 5]. Ventilation with a high driving pressure (Δ*P*), the difference between plateau pressure and positive end-expiratory pressure (PEEP), has been associated with worse outcomes in patients with ARDS [6, 7], and a cut-off value of 15 cm H_2_O for Δ*P* has been proposed and is currently widely used at the bedside as a safety limit.

The pathophysiological mechanisms of VILI remain only partly understood. Complement activation has been suggested as one pathogenetic factor in VILI [8]. However, evidence for a key role of complement activation persists to be poor and originates mainly from preclinical studies. In rats with *Streptococcus pneumoniae* pneumonia, ventilation with a high *V*_T_ increased pulmonary levels of the complement activation product C4b/c [9]. In healthy mice, ventilation with a high *V*_T_ resulted in increased complement C3 deposition in the lung and reduced cell aggregation [10]. In healthy rats, ventilation with a high *V*_T_ increased pulmonary vascular permeability, a finding that was linked to increased plasma levels of soluble terminal complement complex [11]. Also, in a study with healthy rats, ventilation with high airway pressures resulted in increased C3a levels in plasma [12]. Studies in the human setting are even more scarce, and findings in these studies are conflicting with those in preclinical studies. For instance, no association was found between complement depositions in lung tissue and ΔP in critically ill patients who died under invasive ventilation [13].

We initiated the current study to gain a better understanding of the effects of *V*_T_ and Δ*P* on complement activation in the pulmonary compartment of critically ill patients under invasive ventilation. The hypothesis was that pulmonary complement activation is associated with *V*_T_ and Δ*P*, and also that pulmonary levels of complement activation products are higher in patients with ARDS or with pneumonia.

## Methods

### Study design and ethical concerns

This was a sub-study of the prospective observational ‘Biomarker Analysis in Septic Intensive Care patients’ (BASIC) study. BASIC was a single-center investigation performed in the intensive care unit (ICU) of the Amsterdam University Medical Centers, location ‘AMC’, Amsterdam, The Netherlands. The study protocol of BASIC was approved by the Institutional Review Board (METC 2010_335#B201112). The study was registered at the Dutch Central Commission for Human bound Research (CCMO) (study identifier NL34294.018.10). Patients were included from April 2011 to November 2013. Written informed consent was obtained from all patients or their next of kin, before study entry.

### Patients

Patients were eligible for participation in BASIC if: (a) aged 18 years or older; (b) expected ICU stay of at least 24 h; and (c) having at least two criteria for systemic inflammatory response syndrome (SIRS) [14], with or without infectious causality. Readmitted patients, patients who were referred to the participating ICU from an ICU in another hospital, patients treated with antibiotics for > 48 h, patients included in other studies testing interventions that could possibly affect inflammatory processes, and patients of whom no written informed consent was obtained were excluded. For the current analysis, we included patients who were under invasive ventilation and were subjectable to a bronchoalveolar lavage (BAL) (see below). Patients were also excluded if there was no blood sample taken at the moment of the BAL, or if BAL fluid (BALF) was of insufficient quality (see below for definition).

### Clinical data and ventilator variables and parameters

A dedicated team of trained researchers collected baseline characteristics and outcomes, and scored presence of ARDS according to the Berlin definition for ARDS [15]. Presence of pneumonia was assessed using the Centers for Disease Control and Prevention and International Sepsis Forum consensus definitions [16]. Patients with pneumonia on admission and patients having pneumonia within 48 h of start of ventilation, were considered pneumonia patients.

Granular ventilation data were collected from the electronic patient data monitoring system that recorded and stored (a) ventilation mode, (b) expired *V*_T_, (c) PEEP and (d) maximum, peak and plateau airway pressures, and (e) fraction of inspired oxygen (FiO_2_) every 5 min.

### BAL

Within 48 h after ICU admission a miniature BAL was performed as described before [17]. In short, a 50-cm 14-gauge tracheal suction catheter was inserted via the orotracheal tube and advanced until significant resistance was encountered. Then 20 ml of sterile normal saline was instilled over a period of 4 to 5 s. Immediately hereafter, fluid was aspirated, typically recovering 4 to 8 ml. This BALF was processed directly and centrifuged at 1.500×*g* for 15 min at 4 °C; samples were stored at − 80 °C until assays were performed batchwise.

### Assays

The following complement activation products were measured in BALF to determine complement activation. C5a was measured using a commercial enzyme-linked immunosorbent assays (MicroVue, Quidel, San Diego, CA). C3b/c and C4b/c were measured using home-made enzyme-linked immunosorbent assays (Sanquin, Amsterdam, The Netherlands) as described before [18, 19]. As these assays do not distinguish, respectively, C3b from C3bi and C3c, and C4b from C4bi and C4c, we referred to these as C3b/c and C4b/c.

Urea levels were determined in BALF and plasma using quantitative colorimetric urea determination (BioAssay Systems, Hayward, CA).

### Primary and secondary endpoints

The coprimary endpoint was the association between complement activation product C5a in BALF and median *V*_T_ and median Δ*P* from start of invasive ventilation till the BAL.

Other endpoints were the association between pulmonary levels of C5a and median *V*_T_ and Δ*P* in the 6-h time-frame before BAL, associations between other complement activation products and these two ventilator settings, and local levels of C5a in patients with ARDS versus patients without ARDS, and in patients with pneumonia versus patients without pneumonia.

### Power calculation

Data on which we could base a power calculation were lacking. Therefore, we used samples from all patients who were included in the BASIC study, who were under invasive ventilation and underwent a BAL within 48 h after its initiation. With 72 analyzable patients, we have a two-sided significance level of 0.05 and a power of 80% for correlation coefficient (*r*) as low as 0.325.

### Analysis plan

Continuous variables were presented as median (25th–75th interquartile range [IQR]) or mean with standard deviation (SD), where appropriate. Categorical variables are shown in proportions (%). Continuous variables were analyzed using a Mann–Whitney *U*-test or Student’s *t*-test according to data distribution, proportions were compared using a Fisher exact test. From the available 5-min ventilation data, first the median Vt, Δ*P* and PEEP level was calculated for each patient. Then these medians were used to calculate the median and IQR of the whole group. *V*_T_ was expressed in ml/kg predicted bodyweight (PBW) [20]. Δ*P* was calculated by subtracting PEEP from the maximum airway pressure, as all patients were under pressure-controlled modes of ventilation and no plateau airway pressures were available [21, 22].

To correct BALF levels of C5a, C3b/c, and C4b/c for differences caused by dilution of the samples, the ratio between urea in BALF and urea in plasma was used to correct complement concentrations in BALF, as described before [23]. Patients with ‘low quality’ BALF, defined as samples with urea levels below the detection limit, were excluded from the analyses. Levels were presented for the whole group, and for patients with ARDS or pneumonia. Levels were individually plotted on a log-scale together with Tukey boxplots.

The association between levels of pulmonary complement activation and *V*_T_ and ΔP was analyzed in two ways. First, the association with *V*_T_ and Δ*P* from start of invasive ventilation till BAL was investigated. Also, the association with ventilation in the 6 h before BAL was investigated. We used scatterplots and *r* using Pearson’s/Spearman association method according to data distribution. The complete analysis was repeated to compare the effects of ventilator settings on complement activation products in patients with ARDS versus those without ARDS, and in patients with pneumonia and patients without pneumonia.

Statistical analyses were performed using GraphPad Prism version 8.0.2 (GraphPad software Inc, La Jolla, CA, USA). A *P*-value of < 0.05 was considered statistically significant.

## Results

### Patients

**Fig. 1:**
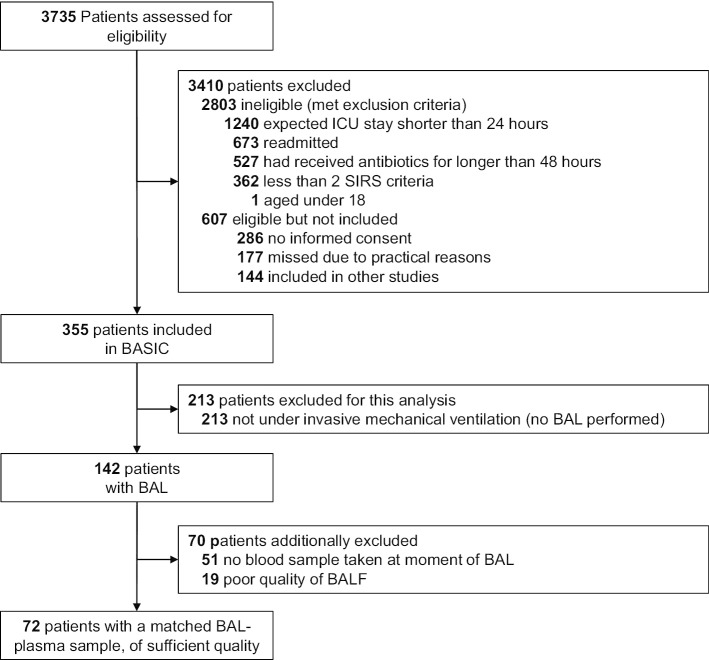
Flow of patients. *ICU* intensive care unit, *SIRS* systemic inflammatory response syndrome, *BASIC-study* ‘Biomarker Analysis in Septic Intensive Care patients’-study, *BAL* bronchoalveolar lavage, *BALF* bronchoalveolar lavage fluid

Flowchart of patients is shown in Fig. 1. In total, 355 patients were included in the BASIC study. After excluding patients who were not under invasive ventilation, patients who did not have a matched BALF to plasma sample, and patients in whom BALF was considered of poor quality, 72 patients remained for the current analysis. Of these patients, 21 patients (29%) were classified as having ARDS, and 29 patients (40%) had pneumonia. Patient characteristics are presented in Table 1. ICU mortality rate was 25%.

**Table 1:** Patient characteristics

	Patients (*N* = 72)	
Age (years), median [IQR]	62.0	[49.3–72.8]
Female gender, *n* (%)	32	(44.4)
Weight (kg), median [IQR]	80.0	[65.0–90.0]
Length (cm), median [IQR]	175.0	[165.3–180.0]
BMI (kg/m^2^), median [IQR]	25.6	[22.7–29.7]
ICU admission type		
Medical, n (%)	58	(80.6)
Surgical emergency, n (%)	10	(13.9)
Surgical elective, n (%)	4	(5.6)
Severity of illness		
Max SOFA score, median [IQR]	10.0	[8.0–13.0]
APACHE IV score, median [IQR]	84.5	[67.0–107.0]
SAPS, median [IQR]	54.5	[47.0–70.3]
Lung injury at start of ventilation		
ARDS, n (%)	21	(29.2)
Pneumonia, n (%)	29	(40.3)
Lowest PaO_2_:FiO_2_, median [IQR]	172	[145–220]
Comorbidities		
COPD, n (%)	4	(5.6)
Sepsis, n (%)	43	(58.3)
ICU outcomes		
ICU length of stay (days), median [IQR]	6.5	[4.0–10.0]
Total MV days, median [IQR]	4.0	[2.8–8.3]
ICU mortality, n (%)	18	(25.0)

*BMI* body mass index, *SOFA* Sequential Organ Failure Assessment, *APACHE* Acute Physiology and Chronic Health Score, *SAPS* Simplified Acute Physiology Score, *ARDS* acute respiratory distress syndrome, *COPD* chronic obstructive pulmonary disease

Median time from start of invasive ventilation to BAL was 27 [IQR 19 to 34] hours. Median *V*_T_ from start of ventilation to BAL was 6.7 [IQR 6.1 to 7.6] ml/kg PBW, comparable to the median *V*_T_ of 6.8 [IQR 6.1 to 8.0] ml/kg PBW in the last 6 h before the BAL. Median ΔP from start of ventilation to BAL was 15 [IQR 11 to 18] cmH_2_O, slightly higher than the median ΔP of 13 [IQR 9 to 18] cmH_2_O in the last 6 h before BAL. Median PEEP from start of ventilation to BAL was 7.8 [IQR 5.0 to 10.0] cmH_2_O, higher than the median PEEP of 6.0 [IQR 5.0 to 10.0] cmH_2_O in the last 6 h before BAL.

### BALF levels of complement activation products

**Fig. 2:**
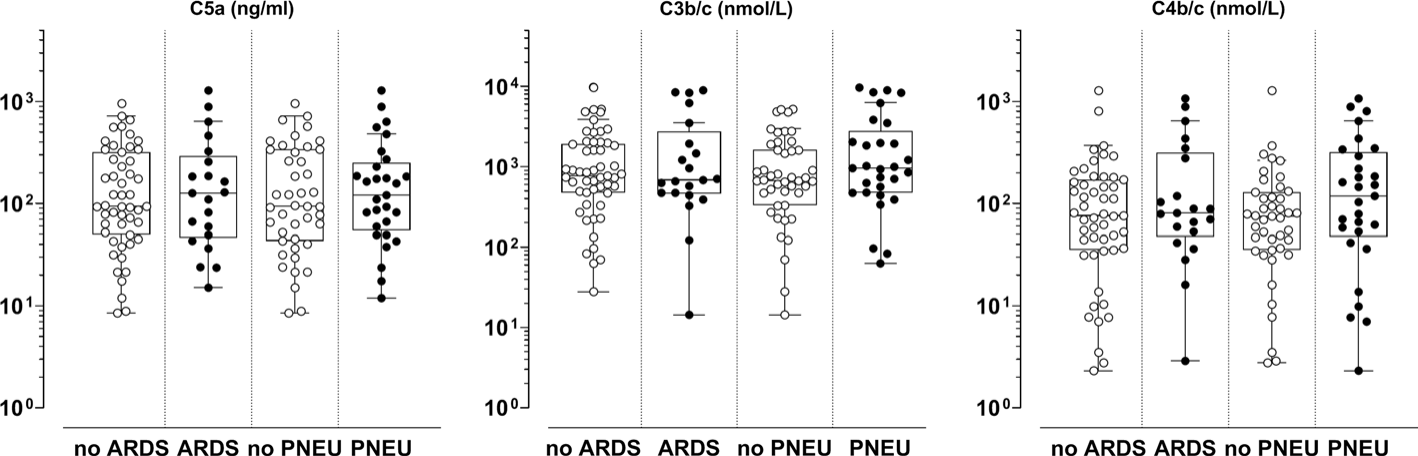
Complement activation products C5a, C3b/c and C4b/c in BALF of patients without ARDS (open symbols) compared to patients with ARDS (closed symbols); and patients without pneumonia (open symbols) and with pneumonia (closed symbols). Individual data were plotted on a log-scale together with Tukey box plots. *ARDS* acute respiratory distress syndrome, *C* complement, *BALF* bronchoalveolar lavage fluid

Median levels of complement activation products in BALF are shown in Fig. 2. Median C5a level was 103 [IQR 49–307] ng/ml, median C3b/c and C4b/c levels were 739 [IQR 471–1939] and 79 [IQR 38–179] nmol/L, respectively. Complement activation products in BALF were similar in patients with ARDS or without ARDS. The same was found for patients with or without pneumonia.

### *Correlations between pulmonary complement activation and V*_*T*_* and ΔP*

**Fig. 3:**
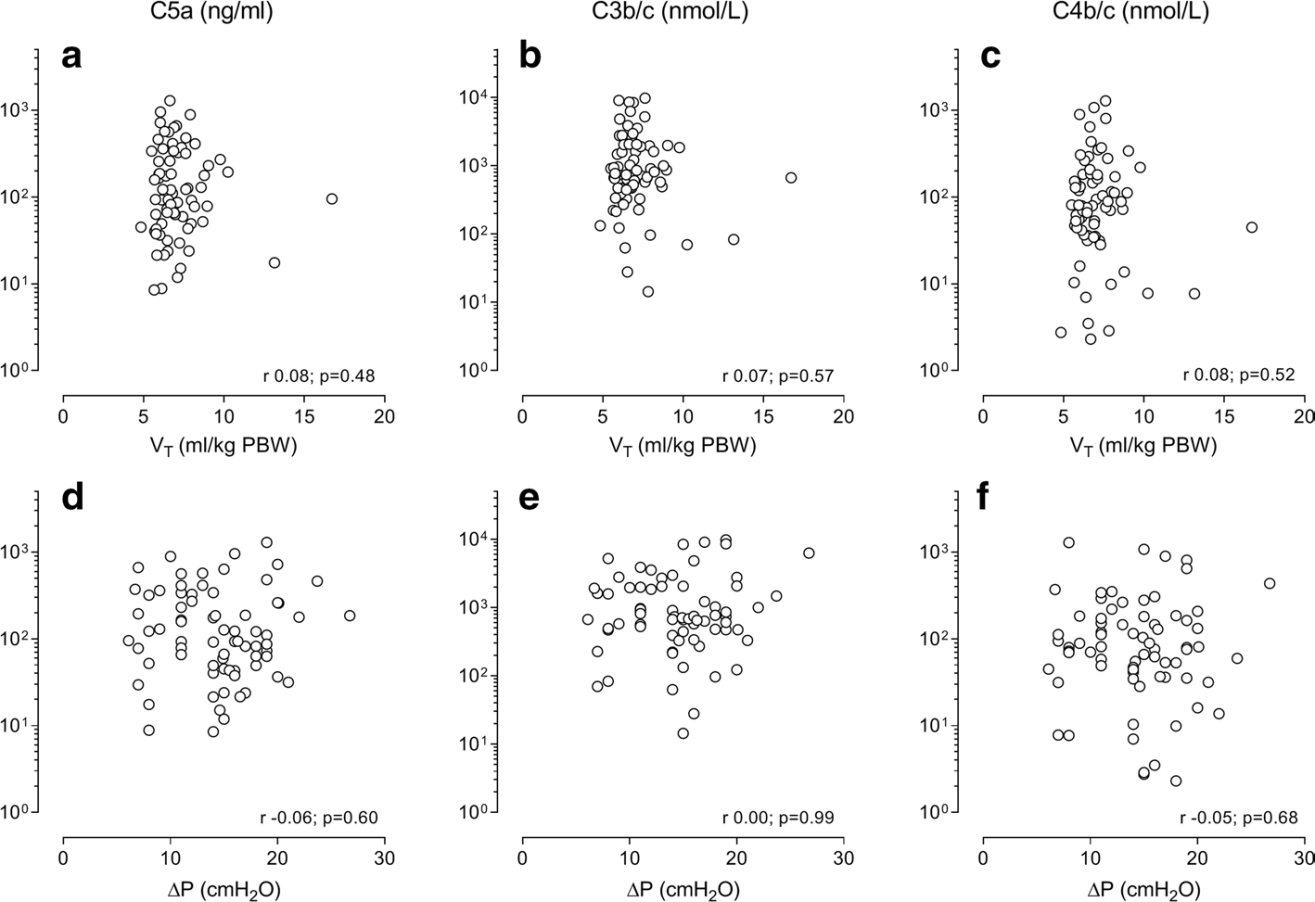
Associations between complement activation products C5a, C3b/c and C4b/c in BALF and *V*_T_ and Δ*P* used prior to BAL. *C* complement, *V*_T_ tidal volume, Δ*P* driving pressures, *BALF* bronchoalveolar lavage fluid

The correlation between BALF levels of complement activation product C5a and *V*_T_ and Δ*P* was poor, as illustrated in Fig. 3. Correlations were also poor between BALF levels of the other two complement components and median *V*_T_ and median Δ*P*.

Restricting the analysis to ventilation data collected within the last 6 h before the lavage, did not change the results (see Additional file 1: Figure S1). Comparing associations between *V*_T_ and Δ*P* in patients with ARDS versus patients without ARDS, and in patients with or without pneumonia resulted in comparable findings (see Additional file 2: Figure S2 and Additional file 3: Figure S3).

## Discussion

The results of this study in a cohort in critically ill patients under invasive ventilation for various reasons can be summarized as follows: (a) no association was found between pulmonary levels of complement activation products and two main ventilator settings, *V*_T_ and Δ*P*, and (b) this was neither different for patients with ARDS and patients without ARDS, nor for patients with pneumonia and patients without pneumonia; (c) one other salient finding was that pulmonary levels of complement activation products were similar between patients with and patients without ARDS or pneumonia.

This study knows several strengths. Bias was minimized by its prospective character, and a preplanned analysis plan was strictly followed. The clear inclusion and exclusion criteria led to a recognizable population of critically ill patients with high severity of illness scores. The number of included patients was large, exceeding the numbers of several preceding studies. Patients underwent a BAL relatively soon after start of invasive ventilation. Last but not least, the electronic patient data monitoring system allowed us to use granular ventilation data, providing an accurate reflection of ventilator settings before BAL in all patients.

Since complement factors are unstable, we payed extensive attention to proper handling of the BAL samples. Samples were immediately processed and stored. All analyses were performed batchwise. Therefore, an effect of complement instability on our results is very unlikely. With a miniature BAL, differences in dilution can result in erroneously low levels of biomarkers of interest, in this case complement activation products. Differences in urea concentration in BAL and plasma were used to correct for this inaccuracy [23]. We improved the quality of our data collection by removing samples from the final analysis when BALF urea levels suggested too much dilution.

Though no data exist on levels of complement activation products in BALF in humans with VILI, several studies have been performed in patients with ARDS [24–26]. One of these studies used C5a as the marker of complement activation, alike we used in the current investigation [24]. In that study, median levels of C5a in BALF were ~ 400 ng/ml, fourfold higher than the median level found in our study. It should be noted, though, that there were important differences in sampling (a miniature BAL in the current study vs a formal BAL in the previous one) as well as sample handling (no further processing in the current study versus concentrating supernatant using pressure filtration. Last but not least, the two investigations used different assays for C5a measurements.

The results of the current investigation are in contrast with findings in previous animal studies [9–12]. In rodent models of VILI, ventilation with a high *V*_T_ [9–11] or a high ventilation pressure [12] resulted in clear complement activation, seen the increased levels of complement activation products. It must be mentioned, that in those studies VILI was induced by ventilation with a high *V*_T_ (12 ml/kg) [9] to an extreme high *V*_T_ (35 ml/kg [10] or 40 ml/kg [11]), or with ventilation with a high peak pressure, and thus probably a high Δ*P* [12]. Those settings no longer reflect current clinical practice. Indeed, in the current study, *V*_T_ and Δ*P* were all within widely recommended ranges [27, 28].

One other important difference between preceding animal studies and the current human investigation is that in the animal studies identical hits could be used in a well-controlled setting in genetically comparable rodents, while the cohort of patients in the current study was heterogeneous and had a variety of pulmonary hits. But even with use of a relatively large cohort of patients, to correct for these variances, no correlation was found between pulmonary levels of complement activation products and the two ventilator settings of interest.

The findings of the current study are in line with one previous human study of our group. In a series of critically ill patients who died under invasive ventilation, we found no association between complement C3d depositions in lung tissue and Δ*P* in the final hours before death [13]. No difference in deposition of C3d in ARDS patients when compared to patients without ARDS was present. Two other studies showed increased BALF levels of complement activation products in ARDS patients [24, 25]. It must be mentioned that in these studies complement levels in patients were compared to levels in healthy volunteers and postoperative patients, and not to levels in critically ill patients without ARDS. In one other study, a difference in levels of pulmonary complement activation was found between trauma patients who developed ARDS and those who did not, but these differences were only present very early after start of ventilation, i.e., within hours after its initiation [26]. It is possible that we were ‘too late’ to find a difference in complement activation in the current study.

Several limitations need to be mentioned. With the use of medians to reflect *V*_T_ and Δ*P* during invasive ventilation, shorter periods of more injurious ventilator settings could have been missed. As mentioned above, this also means that we cannot exclude a possible effect of (much) higher *V*_T_ and Δ*P* on pulmonary complement activation. Use of miniature BAL might be inferior to a formal BAL. However, previous studies showed no differences between these two techniques with regard to, e.g., counts of colony forming units [17]. Miniature BAL has the advantage of being less invasive and is therefore frequently used in studies as alternative to a formal BAL [29–31]. Finally, complement activation within the pulmonary compartment in critically ill patients is possibly already high at baseline. This may partially mask the effects on complement activation of any ventilator setting.

## Conclusion

In this cohort of critically ill patients under invasive ventilation for various reasons, no association between levels of pulmonary complement activation and *V*_T_ or Δ*P* was found. It could be that pulmonary complement activation does not play a major role in VILI, and not even in lung injury per se. If true, treatment with complement inhibitors may not contribute to a better outcome in critically ill patients under invasive ventilation.

## Supplementary information

**Additional file 1: Figure S1.** Association between complement activation products, C5a (A + D), C3b/c (B + E) and C4b/c (C + F) in bronchoalveolar lavage fluid and tidal volume (A-C) and driving pressure (D-F) in the last 6 h before BAL. Abbreviations: C, complement activation product; V_T_, tidal volume; ΔP, driving pressure.

**Additional file 2: Figure S2.** Association between complement activation products C5a (A + D), C3b/c (B + E) and C4b/c (C + F) in bronchoalveolar lavage fluid and tidal volume (A-C) and driving pressure (D-F) in patients with (closed symbols) and patients without acute respiratory distress syndrome (open symbols). Abbreviations: C, complement activation product; V_T_, tidal volume; ΔP, driving pressures.

**Additional file 3: Figure S3.** Association between complement activation products C5a (A + D), C3b/c (B + E) and C4b/c (C + F) in bronchoalveolar lavage fluid and tidal volume (A-C) and driving pressure (D-F) in patients with (closed symbols) and patients without pneumonia (open symbols). Abbreviations: C, complement activation products; V_T_, tidal volume; ΔP, driving pressure.

## Data Availability

The datasets generated during and/or analyzed during the current study are available from the corresponding author on reasonable request.

## Abbreviations

ARDS: Acute respiratory distress syndrome
BAL: Bronchoalveolar lavage
BALF: Bronchoalveolar lavage fluid
ICU: Intensive care unit
BMI: Body mass index
PBW: Predicted bodyweight
APACHE: Acute Physiology and Chronic Health Evaluation
SAPS: Simplified Acute Physiology Scores
FiO_2_: Fraction of inspired oxygen
PEEP: Positive end-expiratory pressure
SIRS: Systemic inflammatory response
VILI: Ventilator-induced lung injury
*V*_T_: Tidal volume
Δ*P*: Driving pressure
IQR: Interquartile range
SOFA: Sequential Organ Failure Assessment
COPD: Chronic obstructive pulmonary disease
