# Supplementary figures and images for: The effects of tidal volume size and driving pressure levels on pulmonary complement activation: an observational study in critically ill patients
Source: Intensive Care Med Exp. 2020 Dec 18;8(Suppl 1):74. doi: 10.1186/s40635-020-00356-6 (PMC7746430; doi:10.1186/s40635-020-00356-6)

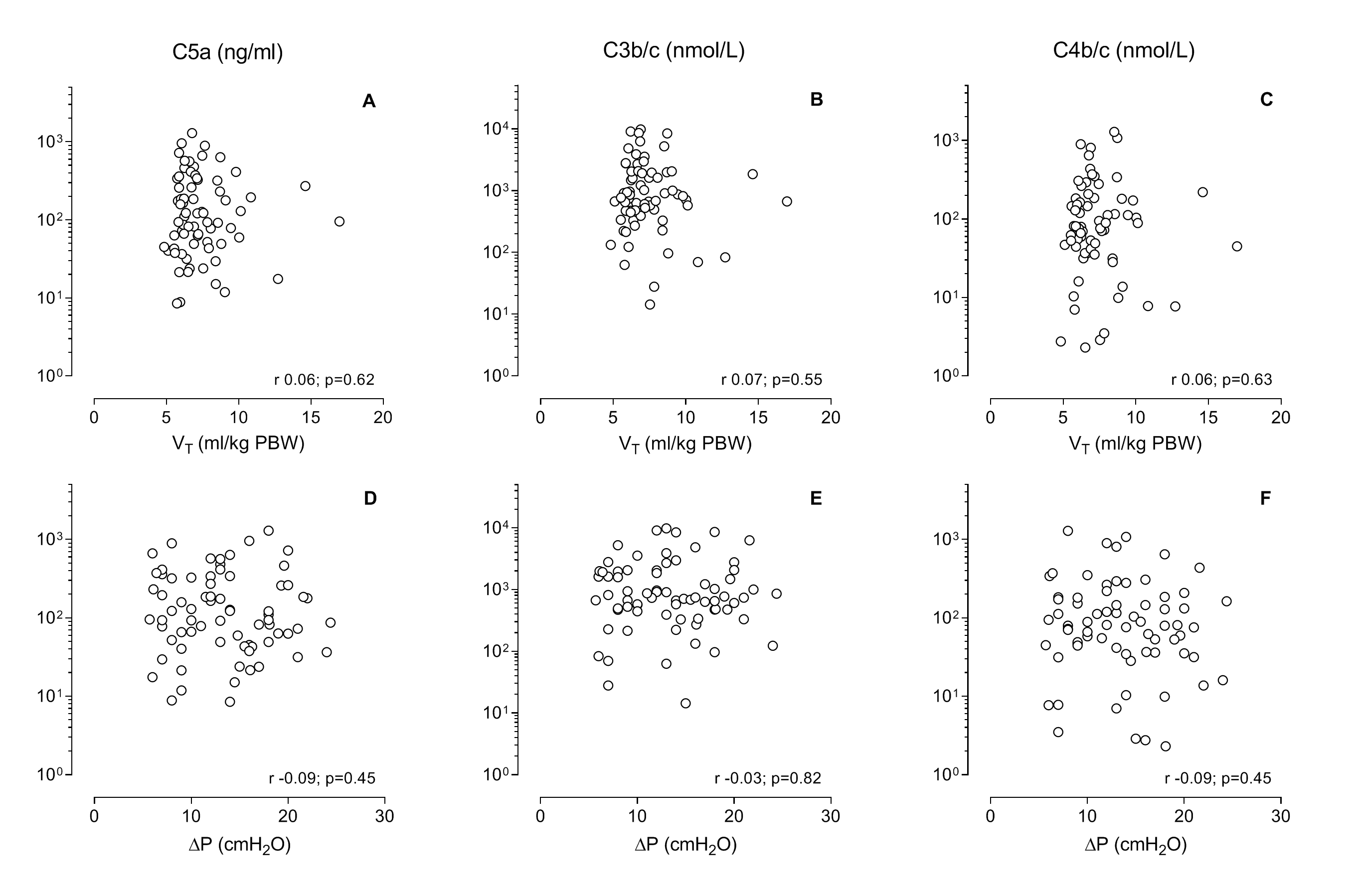

Supplement: Supplementary file 1 — Additional file 1: Figure S1. Association between complement activation products, C5a (A + D), C3b/c (B + E) and C4b/c (C + F) in bronchoalveolar lavage fluid and tidal volume (A-C) and driving pressure (D-F) in the last 6 h before BAL. Abbreviations: C, complement activation product; VT, tidal volume; ΔP, driving pressure. [file 40635_2020_356_MOESM1_ESM.tif]

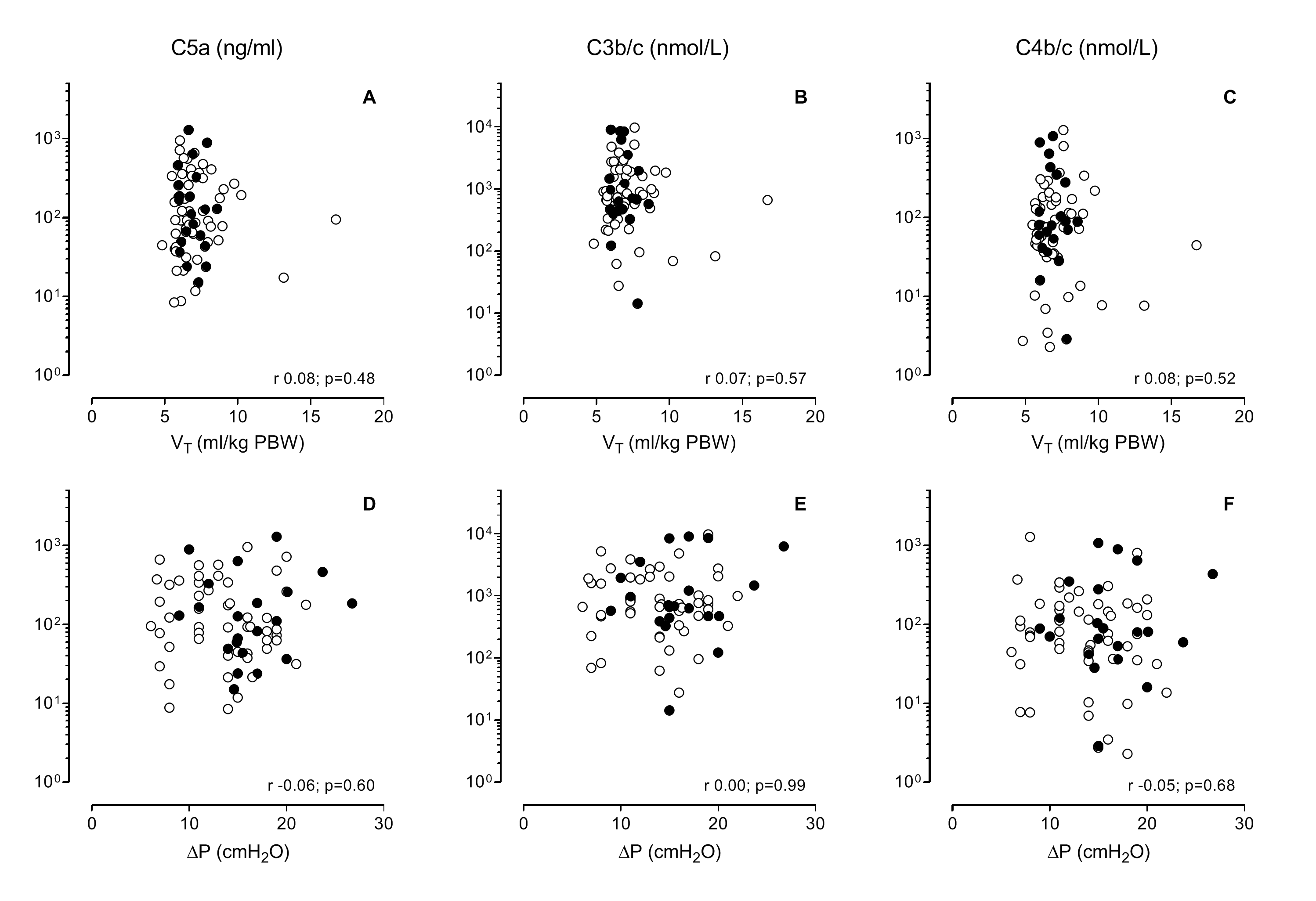

Supplement: Supplementary file 2 — Additional file 2: Figure S2. Association between complement activation products C5a (A + D), C3b/c (B + E) and C4b/c (C + F) in bronchoalveolar lavage fluid and tidal volume (A-C) and driving pressure (D-F) in patients with (closed symbols) and patients without acute respiratory distress syndrome (open symbols). Abbreviations: C, complement activation product; VT, tidal volume; ΔP, driving pressures. [file 40635_2020_356_MOESM2_ESM.tif]

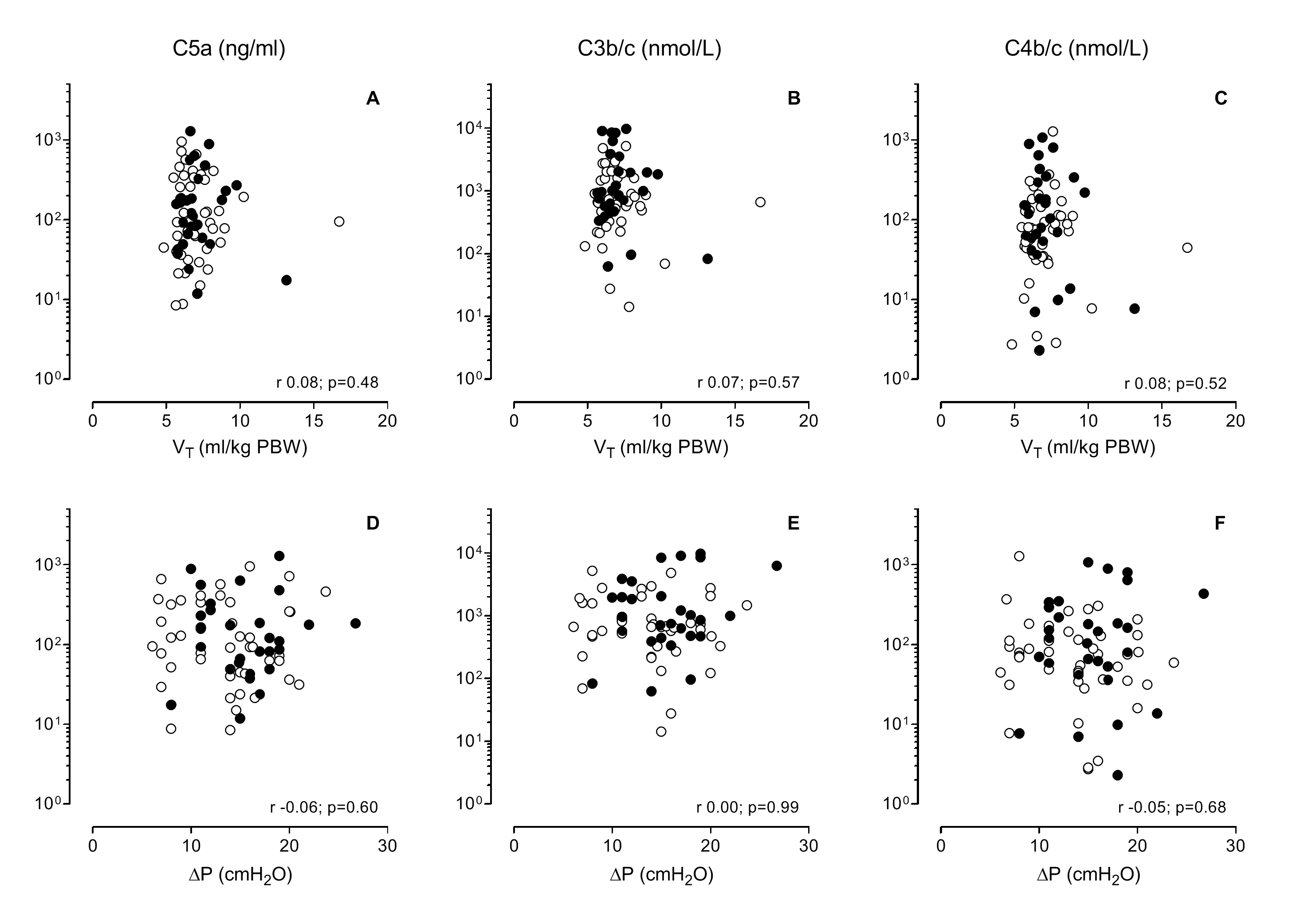

Supplement: Supplementary file 3 — Additional file 3: Figure S3. Association between complement activation products C5a (A + D), C3b/c (B + E) and C4b/c (C + F) in bronchoalveolar lavage fluid and tidal volume (A-C) and driving pressure (D-F) in patients with (closed symbols) and patients without pneumonia (open symbols). Abbreviations: C, complement activation products; VT, tidal volume; ΔP, driving pressure. [file 40635_2020_356_MOESM3_ESM.tif]
